# Integrating water quality index, GIS and multivariate statistical techniques towards a better understanding of drinking water quality

**DOI:** 10.1007/s11356-021-17594-0

**Published:** 2021-12-03

**Authors:** Adil Masood, Mohammad Aslam, Quoc Bao Pham, Warish Khan, Sarfaraz Masood

**Affiliations:** 1 Department of Civil Engineering, Jamia Millia Islamia University, New Delhi, 110025 India; 2 Center of Excellence in Environmental Studies, King Abdulaziz University, Jeddah, 21589 Kingdom of Saudi Arabia; 3 Faculty of Natural Sciences, Institute of Earth Sciences, University of Silesia in Katowice, Będzińska street 60, 41-200 Sosnowiec, Poland; 4 Department of Geography, Jamia Millia Islamia University, New Delhi, 110025 India; 5 Department of Computer Engineering, Jamia Millia Islamia University, New Delhi, 110025 India

**Keywords:** Principal component analysis, Hierarchical cluster analysis, Discriminant analysis, Entropy, Kriging, Semivariogram

## Abstract

Groundwater is considered as an imperative component of the accessible water assets across the world. Due to urbanization, industrialization and intensive farming practices, the groundwater resources have been exposed to large-scale depletion and quality degradation. The prime objective of this study was to evaluate the groundwater quality for drinking purposes in Mewat district of Haryana, India. For this purpose, twenty-five groundwater samples were collected from hand pumps and tube wells spread over the entire district. Samples were analyzed for pH, electrical conductivity (EC), total dissolved solids (TDS), total hardness (TH), turbidity, total alkalinity (TA), cations and anions in the laboratory using the standard methods. Two different water quality indices (weighted arithmetic water quality index and entropy weighted water quality index) were computed to characterize the groundwater quality of the study area. Ordinary Kriging technique was applied to generate spatial distribution map of the WQIs. Four semivariogram models, i.e. circular, spherical, exponential and Gaussian were used and found to be the best fit for analyzing the spatial variability in terms of weighted arithmetic index (GWQI) and entropy weighted water quality index (EWQI). Hierarchical cluster analysis (HCA), principal component analysis (PCA) and discriminant analysis (DA) were applied to provide additional scientific insights into the information content of the groundwater quality data available for this study. The interpretation of WQI analysis based on GWQI and EWQI reveals that 64% of the samples belong to the “poor” to “very poor” bracket. The result for the semivariogram modeling also shows that Gaussian model obtains the best fit for both EWQI and GWQI dataset. HCA classified 25 sampling locations into three main clusters of similar groundwater characteristics. DA validated these clusters and identified a total of three significant variables (pH, EC and Cl) by adopting stepwise method. The application of PCA resulted in three factors explaining 69.81% of the total variance. These factors reveal how processes like rock water interaction, urban waste discharge and mineral dissolution affect the groundwater quality.

## Introduction

Groundwater is a critical freshwater resource for billions of habitants around the world. Its quality and quantity, however, have progressively deteriorated as a result of its intensified anthropogenic exploitation. In light of global changes including meteoric growth of population, unplanned urbanization, industrialization, redundant use of agricultural chemicals and climate change, the groundwater extraction has steadily increased from 312 km^3^/year in the 1960s to 743 km^3^/year in 2000 (Wada et al. 2010; Joarder et al. 2008). During this time frame, the rate of global groundwater loss has increased mostly due to rises in India (23%), China (102%) and USA (31%) (Dalin et al. 2017). Thus, excessive use of groundwater is not only leading to sharp falls in water tables, but also threatening the quality of groundwater resources in many regions across the world.

The quality of groundwater is an essential, critical and equally important factor, as its quantity because it plays a significant role in determining its adequacy for domestic, agricultural and industrial activities. The subsurface hydrogeochemical processes, anthropogenic activities, soil characteristics, seasonal variation, climatic conditions and groundwater recharge are the major factors that influence groundwater quality (Naz et al. 2016; Zhou et al. 2013). In recent years, the deterioration in groundwater quality has increased dramatically due to lack of control over the release of landfill leachate, poor management and other anthropogenic activities causing serious threats to human health (Yadav et al. 2018; Egbueri et al. 2021). A plethora of studies have assessed the groundwater quality by considering various state-of-the-art techniques for different regions across the world. However, most of these techniques have primarily been based on single-parameter assessments, in which governing factors were discretely evaluated and the water quality was largely influenced by the most impaired factor (Şener et al. 2017). Therefore, there is a growing need for techniques which allow better interpretation of water quality in order to ensure effective quality control and management. WQI is generally considered as a reasonable technique which has received high attention from researchers due to its flexibility, adaptability and statistical simplicity to monitor the groundwater quality. The technique helps interpretation of complex water quality data into simple terms (Sadat-Noori et al. 2014).

 A number of researchers have proved the effectiveness of WQI in evaluating water quality for different regions across the world (Machiwal et al. 2018; Duraisamy et al. 2019; Liu et al. 2017). As an example, WQI was developed to study the suitability of groundwater for drinking and agricultural purposes in Malaysia (Harun et al. 2021). From the obtained results, it was concluded that the developed WQI was effective in providing information on the degree of purity and pollution of water in the region. In similar work, an integrated water quality index (IWQI) was developed for evaluating and mapping groundwater quality in Maharashtra, India (Mukate et al. 2019). It was observed that IWQI provided acceptable results for groundwater quality evaluation and may serve as an efficient tool for managing water quality-based health risks.

Conventional methods of groundwater quality assessment have certain limitations in terms of ease of interpretation and depiction of spatio-temporal trends of groundwater. To overcome this problem, numerous water quality indices have been developed and reported in the literature for mapping and evaluating the groundwater quality. For instance, spatio-temporal analysis of the ground water quality in West Bank, Palestine was performed using weighted water quality indices (Judeh et al. 2021). The results of the study indicated that GIS-based water quality index efficiently manages and monitors the trend of changes occurring in the groundwater quality of the region. Similarly, for evaluating the potability of groundwater, a novel GWQI was proposed in order to assess the groundwater quality of Goplaganj district, Bangladesh. WQI-based thematic maps providing spatial variation of ground water quality in reference to potential and vulnerability were generated. It was reported that the spatial distribution of GWQI is a promising technique for gaining good knowledge of groundwater quality conditions within the study area.

The present area under study, located in the Mewat district, is a rural tract, and groundwater remains the primary source of water supply for drinking and agricultural activities. Although a limited number of studies have tried to assess the quality of groundwater in Mewat, e.g. (Mehra et al. 2016; Doley and Sivasami 2003; Sharma 2014), no single research exists which has presented an integrated methodology based on water quality indices, multivariate statistics and geostatistical analysis to characterize the groundwater quality of this region. Thus, there is a research gap in this regard, and more discussion is needed for improved understanding of the degree and sources of groundwater contamination.

Considering all these aspects, a thorough study has been conducted using chemometric data mining techniques (principal component analysis, hierarchical component analysis and discriminant analysis) and geostatistics to elicit the dominant processes influencing the groundwater quality and also show its diversity in spatial extents. Moreover, a couple of water quality indices (GWQI and EWQI) were applied to characterize the groundwater quality with greater precision and provide a general view of its status in the region for drinking purpose.

## Materials and method

### Study area

**Fig. 1:**
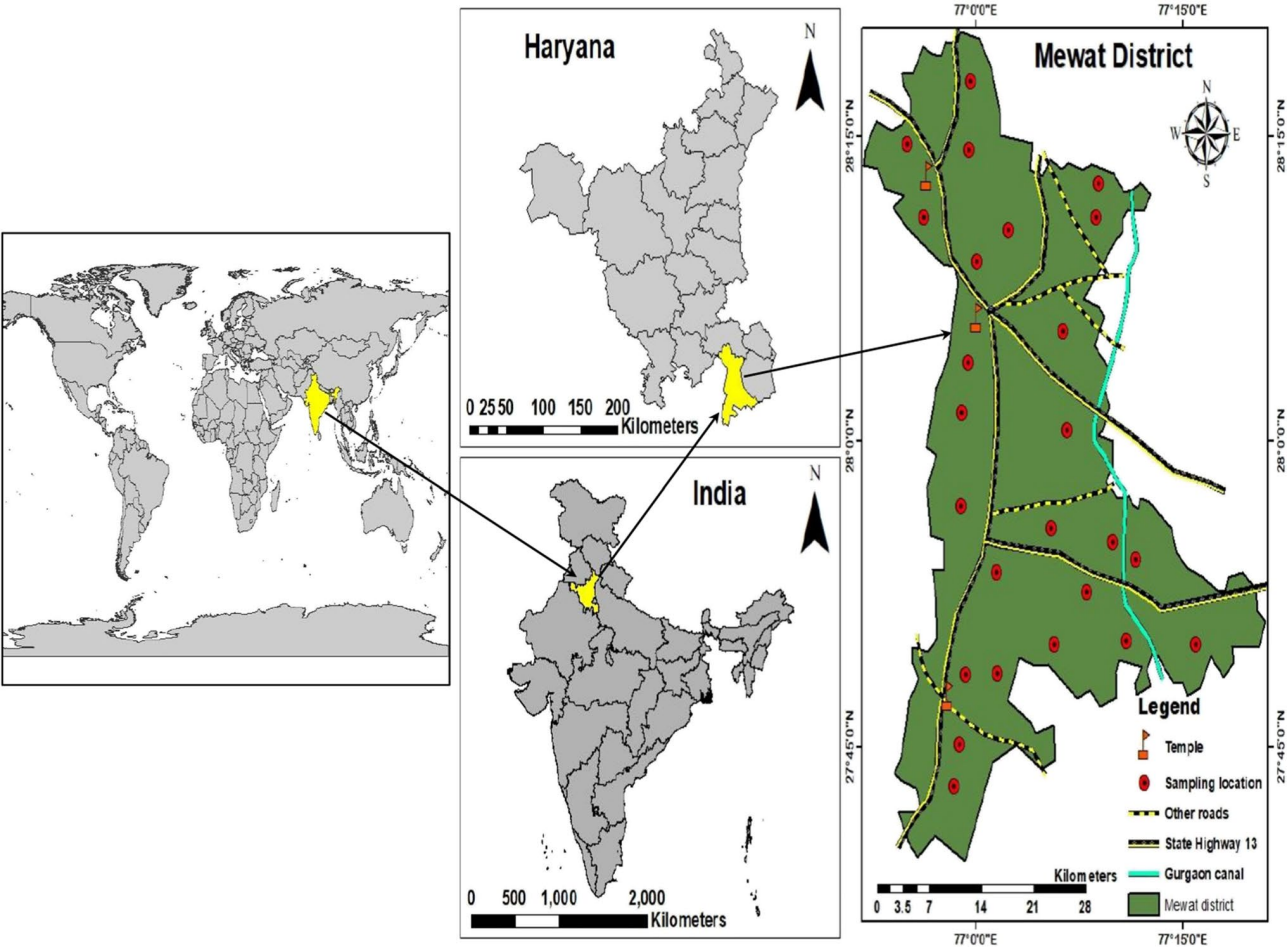
Map of the study area with twenty-five sampling site locations

Mewat district (Haryana) is geographically located between 27°39′ to 28°20′ N and 76°51′ to 77°20′ E. The district covers a total geographical area of 1859.61 km^2^ and is located at a height of 199.49 m above the sea level (Fig. 1). Semi-arid, tropical steppe type hot climatic conditions exist in the district characterized by the extreme dryness of air except during the monsoon months. The mean maximum temperature during the summers is 40 °C, and the mean minimum during the winters is 5.1 °C. The average precipitation in the district (mm/year) is recorded as 594 mm, a significant fraction of which is observed during the course of monsoon season (July–September). The district has an undulating topography with sporadic ridges and hillocks forming a semi circle towards the west, south and east of the Punhana village. The region has a net annual groundwater availability of 21,623Ha-m accompanied by a critical average level of groundwater development of 67%. A high variation in groundwater depth is observed, and the average depth to groundwater table ranges from 4.02±2.75 m (1975) to 10.45±7.55 m (2007) (Mehra et al. 2016).

### Sampling and analysis

Groundwater samples were procured from twenty-five different sites from both hand pumps and tube wells in the month of June (2018), located in Mewat district, Haryana (Fig. 1). The samples were taken after running the water for about 4–6 min in sterilized plastic containers of 1000-ml capacity. These samples had been preserved in airtight ice-cold chests and sent to the testing facility for detailed analysis of various physicochemical parameters using the standard methods (APHA 2005; Egbueri 2020a). Hydrogen ion concentration (pH), total dissolved solids (TDS), electrical conductivity (EC) and turbidity at 25 °C were analyzed in situ using a handheld digital pH/EC/TDS/turbidity meter (HannaHI-9829) respectively. Total hardness (TH), total alkalinity (TA), Cl^−^, Ca^2+^, Mg^2+^, SO_4_^2−^ were all measured following the standard procedures in the laboratory. These parameters were considered based on the expert opinion, data availability and their importance in affecting the groundwater quality. Previous studies have also considered these water quality parameters for examining the groundwater quality trends for different regions across the world (Alfaifi et al. 2020; Solangi et al. 2019; Sengani and Zvarivadza 2018). For the chemical analysis, all reagents utilized were of analytical grade. Double distilled water was used throughout the laboratory testing. The overall quality of sampled groundwater was analyzed using the water quality indices. Two types of indices were developed for this task, i.e. the weighted arithmetic index and the entropy weighted water quality index. MATLAB 9.5 (Mathworks, R2018b) was utilized to compute the water quality indices using the function command. Moreover, ArcGIS 10.1 was used to create and digitize the base map using survey of India topographic sheet (Fig. 1). A number of operations such as data management and editing from the ArcToolbox module were applied. The spatial analyst tool from the toolbox module was used to perform the Kriging technique for generating interpolated maps. The multivariate statistical techniques (DA and PCA) were executed using the IBM SPSS 23, and the HCA was performed in R using the nbCLust package (R Development Core Team 2007).

### Methodology

**Fig. 2:**
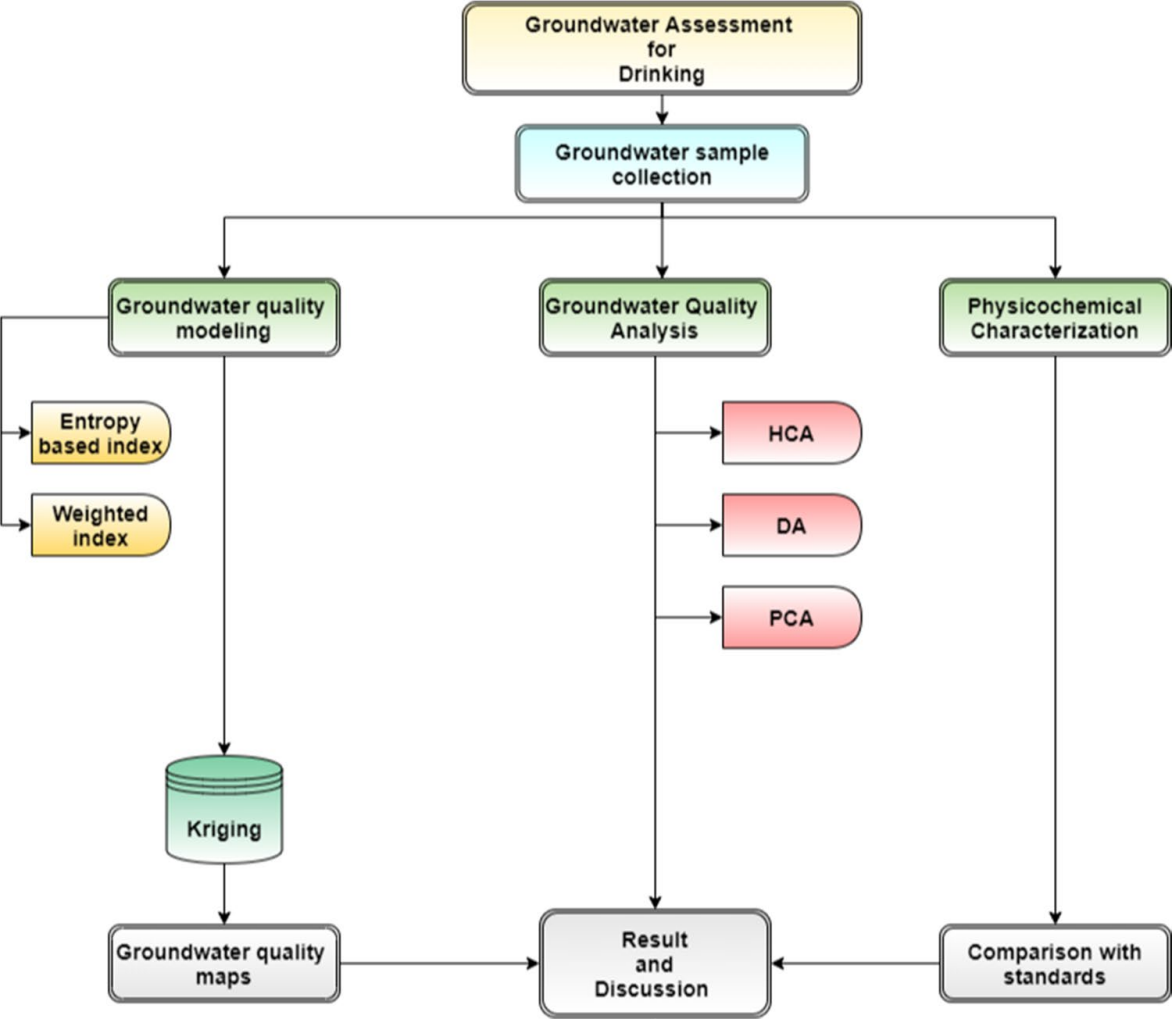
Methodology flowchart for the present study to delineate groundwater quality for drinking purpose

The broad methodological flowchart depicting the various steps involved in groundwater quality evaluation for drinking is shown in Fig. 2. The methodological details for groundwater quality modeling, groundwater assessment mapping and statistical analysis along with their outcomes have been presented in the flow chart.

### Ground water potential modeling

The groundwater quality for this study was modeled using two effective techniques, i.e. weighted arithmetic index approach and the entropy-based water quality index approach. Both of these approaches have been discussed in the following sections.

### Groundwater quality index

The WQI is a dimensionless scale that communicates information on water quality in an enormously easier, lucid and consistent form (Adak et al. 2001; Gupta et al. 2003; Nazir et al. 2016; Mgbenu and Egbueri 2019). For this study, weighted arithmetic index approach has been adopted to perform the quantitative assessments of water quality. To compute the WQI, ten key water quality parameters such as pH, electrical conductivity (EC), total dissolved solids (TDS), chloride, total alkalinity (TA), total hardness (TH), Ca^2+^, Mg^2+^, SO_4_^2−^ and turbidity are taken into consideration. The generic equation for computing GWQI is described as

1 $$WQI=\sum_{i=1}^n{w}_i{q}_i$$

where,

WQI represents a numeric value between 0 and 100; *q*_*i*_ is the water quality score of the *i*^th^ water quality parameter, *w*_*i*_ is the unit weight of the *i*^th^ water quality parameter, *n* represents parameter count.

The quality score *q*_*i*_ is computed using the following relation:

2 $${q}_i=\left[\left({v}_i-{v}_{\ast}\right)/\left({v}_{\mathrm{s}}-{v}_{\ast}\right)\right]100$$

where,

*v*_*i*_ and v_*_ are the true and the ideal values of the *i*^th^ parameter, mostly v_*_ =0, but for certain parameters like pH (v_*_= 7) and DO (v_*_=14.6mg/l). *v*_s_ denotes the standard permissible value for the *i*^th^ parameter. The unit weight *w*_*i*_ is worked out with the equation:

3 $${w}_i=\raisebox{1ex}{$k$}\!\left/ \!\raisebox{-1ex}{${v}_{\mathrm{s}}$}\right.$$

where,

*k* represents the proportionality constant.

The suitability of water quality criteria according to GWQI has been encapsulated in Table 1 below.

**Table 1:** Quality characterization of samples based on GWQI (Alam et al. 2012; Ramakrishnaiah et al. 2009; Ukah et al. 2020)

WQI range	Water quality	Percent of samples	Remarks
<50	Excellent water	28	Beneficial for health and well-being
50–100	Good water	8	Acceptable for potable use
100–200	Poor water	40	Impure and quality unacceptable
200–300	Very poor water	24	Treatment prior to use
>300	Water unsuitable for drinking purposes	0	Needs too much attention

### Entropy-based water quality index

Shannon (1948) originally defined the field of information theory and presented two prime features of this theory in the form of amount of information and Shannon information entropy. In order to calculate EWQI, the following procedure based on Shannon information entropy has been adopted (Amiri et al. 2014; Egbueri et al. 2020). In the initial stage, the entropy weight of each parameter is computed through the following steps:

Let the number of water samples be “*s*” (*i*= 1,2,3,4…*s*) and the number of hydrochemical variables be “*p*” (*j*=1, 2, 3, 4…, *p*).

Subsequently, the Eigen value matrix *X* can be generated using Eq. 4:

4 $$X=\left\{\begin{array}{cccc}{x}_{11}& {x}_{12}& \dots & {x}_{1p}\\ {}{x}_{21}& {x}_{22}& \dots & {x}_{2p}\\ {}\vdots & \vdots & \vdots & \vdots \\ {}{x}_{S1}& {x}_{S2}& \cdots & {x}_{SP}\end{array}\right\}.$$

A normalized matrix, *Y*, is created by applying a normalizing function to eigen value matrix, *X*, in order to eliminate the impact of various units and dimensions of water quality variables. The normalized matrix *Y* is developed as shown below:

5 $$Y=\left\{\begin{array}{cccc}{y}_{11}& {y}_{12}& \dots & {y}_{1p}\\ {}{y}_{21}& {y}_{22}& \dots & {y}_{2p}\\ {}\vdots & \vdots & \vdots & \vdots \\ {}{y}_{S1}& {y}_{S2}& \cdots & {y}_{SP}\end{array}\right\}.$$

The index’s effectiveness of risk for parameter *j* in the sample number *i* is determined using Eq. 6:

6 $${P}_{ij}=\frac{y_{ij}}{\sum_{i=1}^s{y}_{ij}}.$$

The expression used for estimating information entropy (*e*_*j*_) is as follows:

7 $${e}_j=-\frac{1}{{\ln}\ s}\sum_{i=1}^s{P}_{ij}\ln {P}_{ij}.$$

An inferior value of entropy signifies a greater impact of *j* index. The entropy weight (*w*_*j*_) for the variable *j* is computed using Eq. 8.

8 $${w}_j=\frac{1-{e}_j}{\sum_{j=1}^P\left(1-{e}_j\right)}$$

In the next phase, the qualitative ranking criteria (*Q*_*j*_) is determined for every variable using the following equation (Eq. 9):

9 $${Q}_j=\frac{C_j}{S_j}\times 100$$

*C*_*j*_ is the concentration of *j*^th^ parameter in mg/l, and *S*_*j*_ is the Indian standards for groundwater quality in mg/l.

The final phase involves computation of EWQI and is given by the following relation:

10 $$EWQI=\sum_{j=1}^P{w}_j{Q}_j$$

Based on EWQI, the groundwater quality is characterized into five classes as shown in Table 2.

**Table 2:** Quality characterization of groundwater based on EWQI (Jianhua et al. 2011)

EWQI	Rank	Water quality	Percent of samples
<50	1	Excellent	-
50–100	2	Good	-
100–150	3	Medium	36
150–200	4	Poor	36
>200	5	Extremely poor	28

### Hierarchical cluster analysis

Hierarchical cluster analysis (HCA) is a robust data mining technique capable of pattern recognition within homogeneous groups or clusters of cases (variables) (Egbueri 2020b). The fundamental concept that drives this technique is to form a binary data tree that successively merges similar group of points. The emerging groups of points should then display strong intra-cluster homogeneity and a strong inter-cluster heterogeneity (Kazi et al. 2009; Egbueri 2021). These techniques are applied to develop and merge homogeneous group of water samples into significant clusters and ascertain spatial similarity and location clustering within the sampling stations. Moreover, the clustering is accomplished using Ward’s linkage criterion, and the results are illustrated in the form of a 2-D plot called dendrogram.

### Principal component analysis

Principal component analysis (PCA) is an exploratory data analysis technique that is often used to reduce high-dimensional data into a lower dimensional data. The original data set, having many correlated variables, can often be interpreted in just a few uncorrelated variables (axes), known as principal components (PC). These variables are linearly independent (orthogonal) and are a product of original correlated variables with the eigenvectors, which are lists of coefficients (called weightings). The PCs are produced in a sequential array of elements with reducing contribution to the overall variability, i.e. the first PC describes the highest fraction of variance in the dataset, and successive PCs describe the remaining fraction of variance.

### Discriminant analysis

Discriminant analysis (DA) is a regression-based statistical technique that is used to estimate the relationship between several numerical independent variables (also known as discriminatory variables) and a single nominal dependent variable, such as membership in one or two groups. The prime objective of the analysis is to develop discriminant functions that are nothing but linear combination of discriminatory variables which allow discrimination between the categories of the dependent variable in an optimal manner. These weighted linear combinations are referred to as canonical functions. The first canonical function describes the specific linear combination of variables that maximizes the ratio of among group to within group variance in any single dimension. It generates a discriminant function for each group as follows:

11 $$f\left({D}_j\right)={P}_j+\sum_{k=1}^n{w}_{jk}\times {p}_{jk}$$

where *j* is the number of groups (*D*), *P*_*j*_ is a constant inherent to each group, *n* is the number of parameters used to classify a dataset into a given group, *w*_*jk*_ is the weight coefficient assigned by DA to given parameters (*p*_*jk*_) ( Singh et al. 2004).

## Results and discussion

### Physicochemical characterization of groundwater

**Fig. 3:**
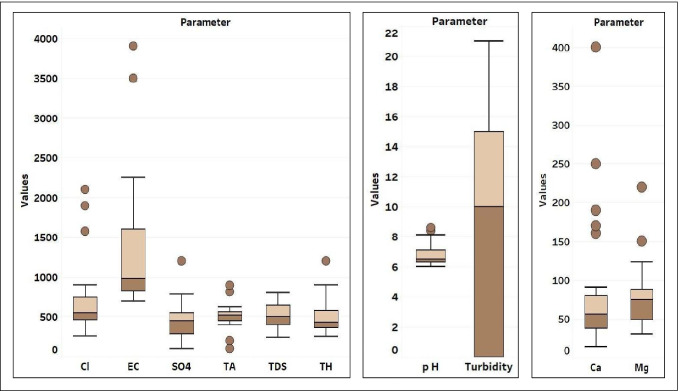
Box plots for the groundwater quality parameters Cl^−^, SO_4_^2−^, Mg^2+^, Ca^2+^, turbidity, total alkalinity (TA), total dissolved solids (TDS), total hardness (TH), electrical conductivity (EC). Units of Cl^−^, SO_4_^2−^, TDS, TH, Ca^2+^, Mg^2+^ are in mg/l; units of EC and turbidity are μs/cm and NTU, respectively

The descriptive statistics of the data for all the 10 physicochemical parameters considered for the groundwater samples and their corresponding permissible limits have been shown in Table 3 and as box plot in Fig. 3. The pH value for our investigation ranges from 6.0 to 8.6 with an average value of 6.77 signifying the slightly acidic nature of the groundwater. In majority of the sites, the pH was within the permissible limit for drinking as specified by WHO (6.5–8.5) apart from the site 23 (8.6).

**Table 3:** Statistical summary of groundwater quality data (*n*=25) and comparison with BIS and WHO standards

Parameters	Mean	Median	SE	SD	Kurtosis	Skewness	Range	BIS (2012)	WHO (2011)
TA (mg/l)	517.04	519`	31.65	158.26	2.60	−0.23	800	200	-
TH (mg/l)	509.40	430	45.52	227.61	2.21	1.48	950	200	100
EC (μs/cm)	1357.48	980	169	845.43	3.42	1.91	3205	-	-
pH	6.77	6.50	0.14	0.72	1.08	1.26	2.6	6.5–8.5	7–8
Cl^−^ (mg/l)	705.20	550	95.28	476.40	3.38	1.95	1841	250	250
SO_4_^2−^ (mg/l)	461.50	450	45.52	227.60	3.54	1.35	1100	200	250
Turbidity (NTU)	9.20	10	1.44	7.19	−1.28	−0.002	21	1	0
TDS (mg/l)	522.76	505	31.61	158.03	−0.99	0.14	563	500	1000
Ca^2+^ (mg/l)	84.52	56	17.60	88.00	6.36	2.41	386	75	75
Mg^2+^ (mg/l)	78.08	75	8.19	40.95	5.21	1.94	190	30	30

*SD* standard deviation, *SE* standard error, *WHO* World Health Organization, *BIS* Bureau of Indian Standards

The electrical conductivity (EC) in the study region exhibits large variations, and its value ranges from 695 to 3900 μs/cm with an average value of 1376 μs/cm. Elevated level of EC in groundwater may be a sign of water circulation, surface infiltration and cation exchanges. On the basis of electrical conductance, groundwater could be ranked into four classes; low conductivity class (EC<500 μs cm^−1^), medium conductivity class I (EC: 500–1000 μs/cm), medium conductivity class II (EC: 1000–3000 μs/cm) and high conductivity class (EC>3000 μs/cm) (Sarma and Swamy 1981). Based on this categorization of EC, 56% of the samples represent Medium class I, 36% of the samples represent Medium class II and rest 8% of the samples relate to high class category. The TDS of the groundwater ranges from 237 to 800 mg/l with an average value of 522.76 mg/l. Water with a TDS concentration below 1000 mg/l is categorized as fresh; 1000–10,000 mg/l as slightly brackish; 10,000–100,000 mg/l as brackish and more than 100,000 mg/l as brine (Todd 1980). All the samples belong to the freshwater category as per the aforementioned classification.

Total hardness (TH) of the samples varied from 250 to 1200 mg/l with a mean value of 509.4 mg/l. As per the classification of TH reported by Sawyer and McCarty (1978), 88% of the samples are categorized as very hard with TH above 300 mg/l, and remaining 12% represent the hard category with TH ranging between 150 and 300 mg/l. Our results are also consistent with those of the study on the Yinchuan Plain, China, which reported similar variation of TH among the analyzed water samples (Liu et al. 2020). Total hardness (TH) of water has no adverse effects on human health, but consumption of hard to very hard water for long periods may induce a high rate of occurrence of urolithiasis, anencephaly, parental mortality, cardiovascular disorders and even cancer in some cases (Durvey et al. 1991).

The chloride concentration of the samples varied from 259 to 2100 mg/l with a mean value of 705.28 mg/l, which indicates pollution and groundwater contamination. All the groundwater samples exhibit chloride concentration greater than the acceptable limit of 250 mg/l (BIS 2012). A similar trend of high chloride concentrations was also reported by El baba et al. (2020) for the groundwater samples of Gaza Strip, Palestine. Chloride in excessive amounts imparts a salty taste to water and increases its corrosivity, and exposure to high chloride levels may cause a laxative effect on humans (Pius et al. 2012; Sadat-Noori et al. 2014).

The minimum and maximum value for SO_4_^2−^ were measured as 100 and 1200 mg/l with a mean value of 461.56 mg/l, in excess of the limits specified by Bureau of Indian Standard (BIS) 10500 ( 2012) of 200 mg/l. Increase in SO_4_^2−^ concentration may be linked to agriculture runoff as the study area has intense agriculture-driven activity. High sulfate concentration in groundwater leads to gastrointestinal irritation and develops a purgative effect on humans (CPCB 2008). Calcium and magnesium levels of the samples vary from 14 to 400 mg/l and 30 to 220 mg/l. The maximum acceptable and permissible limits of Ca^2+^ and Mg^2+^ as prescribed by BIS 10500 ( 2012) for the purpose of drinking are 75–200 mg/l and 30–100mg/l. Ca^2+^ concentration surpassed the permissible limits for drinking water in 8% of the samples, whereas Mg^2+^ was also found in excess of the limits for 16% of the samples. In groundwater generally Mg^2+^ concentration remains less than Ca^2+^, but 44% of the groundwater samples in our study still exhibit high Mg^2+^ concentration over Ca^2+^. A similar cation concentration pattern was reported by Saha et al. (2019) in their study for Rangpur, Bangladesh. Both of these cations contribute to water hardness, and long-term consumption of high Ca^2+^ and Mg^2+^ rich groundwater may result in cardiovascular diseases, reproductive failures, diarrhea and growth retardation (Fatoba et al. 2017) .

### Characterization of groundwater quality based on EWQI and GWQI

**Fig. 4:**
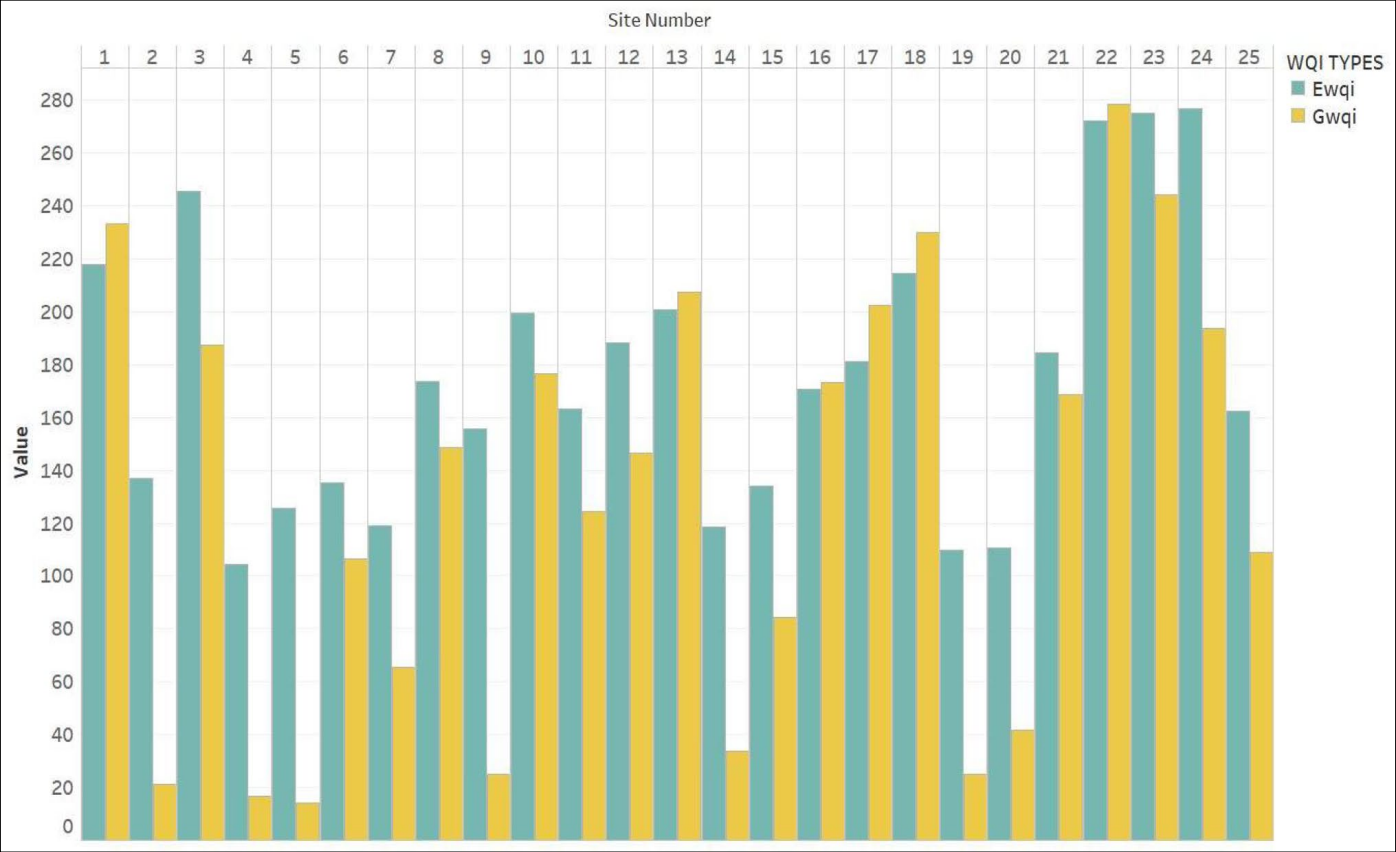
Comparison of GWQI and EWQI based on ground water quality of the study area

The computed results of both EWQI and GWQI methods depict more or less alike trends for majority of the groundwater samples (Fig. 4). As shown in Fig. 4 and Table 2, 28% of the groundwater samples belonging to the sites 2, 4, 5, 9, 14, 19 and 20 had “excellent” water quality based on GWQI-based classification. 8% of the samples representing the sites 7 and 15 showed “good water” quality. 40% of the samples belonging to the sites 3, 6, 8, 10, 11, 12, 16, 21, 24 and 25 indicated “poor water” quality. Unfortunately, 24% of the samples for the sites 13, 17, 18, 22 and 23 had “very poor quality”, and none of the samples represented “water unsuitable for drinking purposes” category. Similarly, 36% of the samples belonging to the sites 2, 4, 5, 6, 7, 14, 15, 19 and 20 had shown “medium” water quality based on EWQI-based classification. Again, 36% of the samples for the sites 8, 9, 10, 11, 12, 16, 17, 21 and 25 indicated “poor” water quality. 28% of the samples representing the sites 1, 3, 13, 18, 22, 23 and 24 showed “extremely poor” quality.

### Geostatistical analysis

In this work, the semivariogram model along with OK (ordinary kriging) has been applied after normalizing the data with log transformation technique. Four semivariogram models, i.e. circular, spherical, exponential and Gaussian were used and found to be the best fit for analyzing the spatial variability in terms of EWQI and GWQI, respectively. These computed semivariogram models along with their characteristics such as nugget, sill, nugget/sill ratio have been presented in Table 4. To gauge the prediction performance of these models, four standard statistical indices, average standard error (ASE), root mean square error (RMSE), mean standardized error (MSE) and root mean square standardized error (RMSSE), were computed. On the basis of the results presented in Table 4, the Gaussian model was considered as the best-fit semivariogram model for both EWQI and GWQI dataset. The RMSE and ASE values of 55.369 and 54.354 for EWQI and 91.43 and 191.829 for GWQI presented by the Gaussian model were the lowest among all other models. In addition to this, the MSE values, which should ideally be zero, were observed as −0.040 for EWQI and 0.023 for GWQI, and the RMSSE values, which should ideally be 1, were observed as 1.049 for EWQI and 0.548 for GWQI, respectively.

**Table 4:** Description of the best-fitted variogram model developed for the groundwater quality parameters

Parameters	Best-fit model	Nugget	Sill	Nugget/ sill	ASE	RMSE	MSE	RMSSE
GWQI	Circular	0.817	1.012	0.807	193.923	91.515	0.025	0.546
	Spherical	0.818	0.989	0.827	194.980	91.52	0.025	0.545
	Exponential	0.819	0.957	0.855	197.979	91.51	0.031	0.532
	Gaussian	0.835	1.035	0.806	191.829	91.43	0.023	0.548
EWQI	Circular	0.081	0.097	0.835	54.696	55.801	−0.042	1.062
	Spherical	0.081	0.095	0.852	54.79	55.779	−.0419	1.060
	Exponential	0.083	0.092	0.902	55.366	55.482	−0.46	1.050
	Gaussian	0.082	0.101	0.811	54.354	55.369	−0.040	1.049

*RMSE* root mean square error, *MSE* mean standardized error, *RMSSE* root mean square standardized error, *ASE* average standard error

**Fig. 5:**
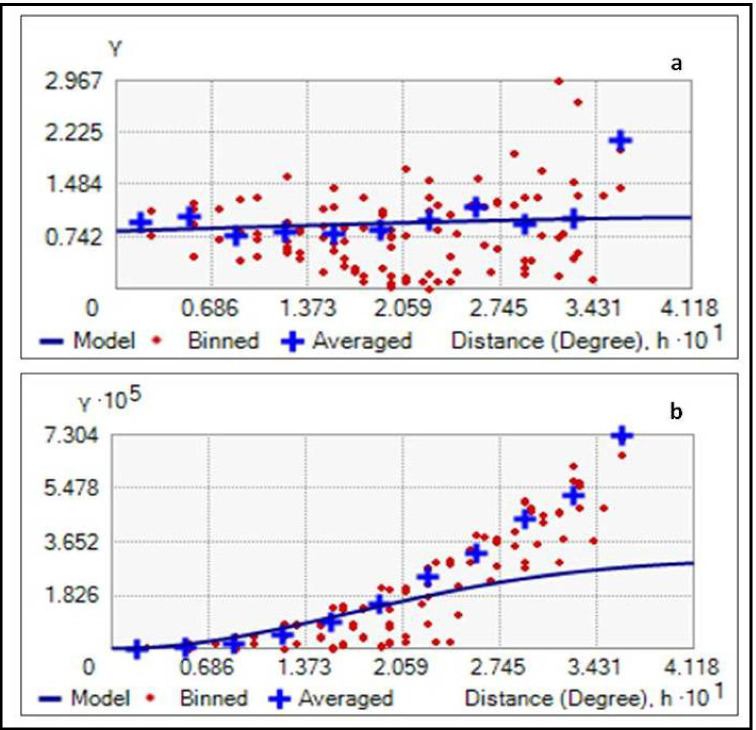
The best-fit semivariogram models for **a** GWQI and **b** EWQI

Similar performances of the semivariogram models have also been reported during a study based on the groundwater quality assessment of Sylhet district, Bangladesh (Islam et al. 2017). The spatial dependence of the groundwater quality indices is generally represented by the nugget/sill ratio. In our study, the nugget/sill values of 0.811 and 0.806 have been obtained for both the indices. In terms of standard classification, a value of the nugget/sill ratio (>0.75) is considered as high and indicates a strong spatial dependence; a value between 0.25 and 0.75 suggests moderate correlation, and a lower value (<0.25) implies a poor (weak) spatial correlation. Moreover, in the case of semivariogram model (Fig. 5a, b), both the indices presented a weak spatial dependence which strongly indicates a role of natural and anthropogenic factors in spatially affecting the groundwater quality.

**Fig. 6:**
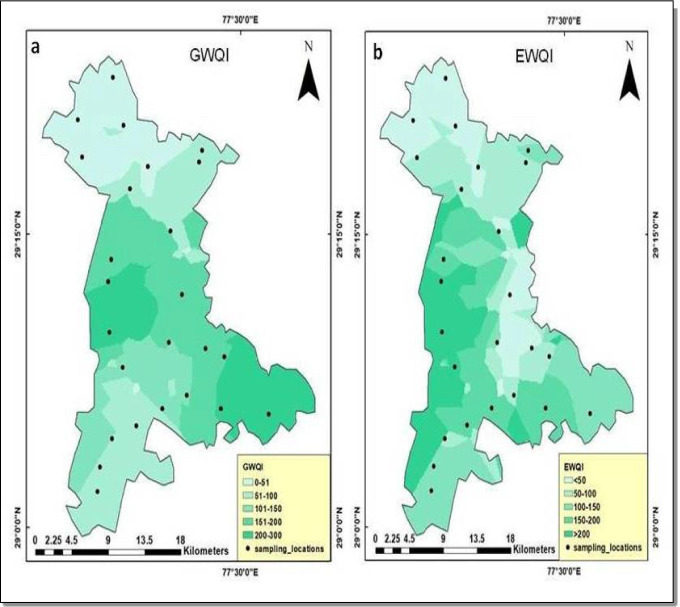
Spatial distribution maps based on ordinary kriging (OK) for **a** GWQI and **b** EWQI for Mewat region

The EWQI and GWQI variability thematic maps were prepared for the study area using the universal kriging technique (Fig. 6a, b). An almost identical spatial distribution pattern was reported in both the indices. The spatial map of GWQI values depicts the grade of water quality in distinct color codes (light green to dark green) (Fig. 6a). Low scores of GWQIs (0–50) representing excellent water quality are observed towards the northern and southern parts of the study region, and poor to very poor quality values (200–300) are observed across much of the midwestern, central and southeastern parts. The moderate values in the range of 100–200 are largely widespread, not confined to any particular portion of the research locale. The GWQI for the study region varies from 21 to 278 with a mean value of 130 (Fig. 4). A major portion of the GWQI map represents poor to very poor grade of groundwater quality. This observation may be an outcome of the indecorous management of domestic, agricultural and industrial effluents in the region. The spatial map of EWQI values also represents the grades of groundwater quality as shown in Fig. 6b. The calculated EWQI values for the district range from 104 to 276 with an average value of 175 (Fig. 4). The map showed that the low values (<50) of EWQI representing excellent water quality category are observed towards the northern and mideastern part of the study region, and the values (100–150) representing the moderate category are largely found in the northern, northeastern and southeastern regions of the study region. Moreover, EWQI values representing the poor and the very poor category (150–200; >200) are widespread and not confined to any portion of the map and are largely observed in the midwestern and southwestern sections of the research locale. It is apparent from the above discussion that both the indices prove to be efficient probabilistic indicators of groundwater quality variation, and the slight difference observed in the results is due to the difference in weighting procedures adopted for computing these indices.

### Multivariate statistical analysis

**Fig. 7:**
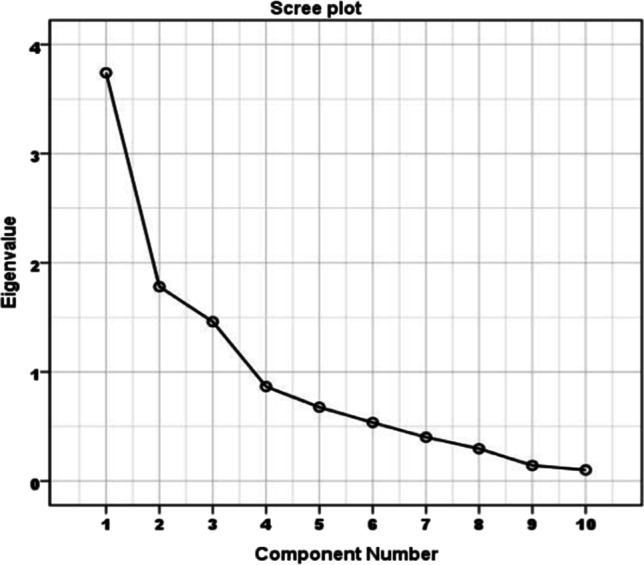
Scree plot of the Eigen values of principal components

For PCA, the Kaiser–Meyer–Olkin (KMO) and the Bartlett test of sphericity have been conducted to determine the sampling adequacy. The KMO measure value of 0.6 has been achieved which is acceptable (KMO >0.49) and is interpreted as mediocre in terms of the degree of common variance. Bartlett’s sphericity test on the correlation matrix of variables is significant and displays the computed *χ*2=104.26 (*p* = 0.000001 and degrees of freedom = 45), implying that PCA can optimally reduce dimensionality of the original unsupervised dataset (Li et al. 2020). Moreover, R-mode PCA is applied on the dataset belonging to the 25 groundwater sampling locations as shown in Table 5. A scree plot (Fig. 7) has been generated to ascertain the number of PCs taken, in order to decipher the underlying data structure (Helena et al. 2000). As shown in Table 6, three PCs based on eigen value greater than 1 are rendered which explain 69.81% of the total variance of the dataset (Hatvani et al. 2018).

**Table 5:** Principal component analysis: varimax rotated R-mode loadings, communalities and percentage of loading matrix

Parameters	PC1	PC2	PC3
R mode			
TA	−0.429	−0.680	0.259
TH	0.808	0.243	0.300
EC	0.835	−0.004	0.600
pH	0.761	0.165	−0.262
Cl^+^	0.827	0.093	0.298
SO_4_^2−^	0.389	−0.063	0.066
Turbidity	0.036	−0.089	0.869
TDS	0.135	0.715	0.120
Ca^2+^	0.248	0.805	0.258
Mg^2+^	0.182	0.411	0.686
Eigen value	3.74	1.78	1.46
Variability (%)	30.61	22.91	16.29
Cumulative (%)	30.61	53.52	69.81

**Table 6:** Principal component analysis: varimax rotated Q-mode loadings, communalities and percentage of loading matrix

Sites	PC1	PC2	PC3
Q mode			
S1	0.180	0.578	0.718
S2	0.259	0.492	0.825
S3	0.309	0.161	0.858
S4	0.504	0.492	0.686
S5	0.523	0.551	0.600
S6	0.774	0.544	0.243
S7	0.829	0.474	0.234
S8	0.813	0.539	0.195
S9	0.832	0.441	0.317
S10	0.712	0.381	0.576
S11	0.220	0.642	0.683
S12	0.770	0.535	0.331
S13	0.410	0.668	0.590
S14	0.518	0.671	0.514
S15	0.504	0.653	0.541
S16	0.573	0.670	0.410
S17	0.495	0.769	0.343
S18	0.590	0.738	0.312
S19	0.305	0.861	0.305
S20	0.438	0.812	0.362
S21	0.795	0.179	0.409
S22	0.374	0.343	0.843
S23	0.878	0.148	0.445
S24	0.801	0.360	0.370
S25	0.500	0.210	0.805

In this study, the first factor PC1, which accounted for 30.61% of the overall variance, showed strong positive loadings for TH, EC, pH and Cl^−^. The factor was significantly distributed in S6–S10, S12, S21, S23 and S24 sample locations (Table 6). Strong loading on EC indicated that the rock-water interaction has promoted active participation of dissolved ions in groundwater (Batabyal and Chakraborty 2015). Similarly, prominent loading on Cl^−^ signified point source pollution through urban waste discharge (Usman et al. 2014), and a high score of pH may be attributed to the mineral dissolution-based reaction with soil CO_2_ (Machiwal and Jha 2015).

The outcome for the first factor PC1 is consistent with those of the study on the Noakhali District, Bangladesh, which reported similar percentage variance with strong positive loading on Cl^−^ , EC, TH, NO_3_^−^ and SO_4_^2−^ (Islam et al. 2021). The second factor, i.e. PC2 exhibited 22.91% of the overall variance which showed strong positive loading on Ca^2+^ and moderate positive loading on TDS. Additionally, the component showed negative loadings on TA and SO_4_^2−^. These loadings were distributed in S13–S20 sample locations. It was observed that this factor probably reflected geogenic activity in the aquifer system and revealed ionic or reverse ionic exchange which ultimately affects the groundwater quality (Bhuiyan et al. 2016).

Similar results for the second factor PC2 were also reported by Omonona et al. (2014) for the groundwater samples of Enugu metropolis, Nigeria.

The third factor PC3 explained 16.29% of total variance with strong absolute positive loading for turbidity and moderate loading for Mg^2+^ in S1–S5, S11, S22 and S25 sampling locations. It was observed that strong loading factor of turbidity indicated the presence of suspended particulates resulting from organic, inorganic and other microbial contaminants present in the water samples (Pant 2011).

**Fig. 8:**
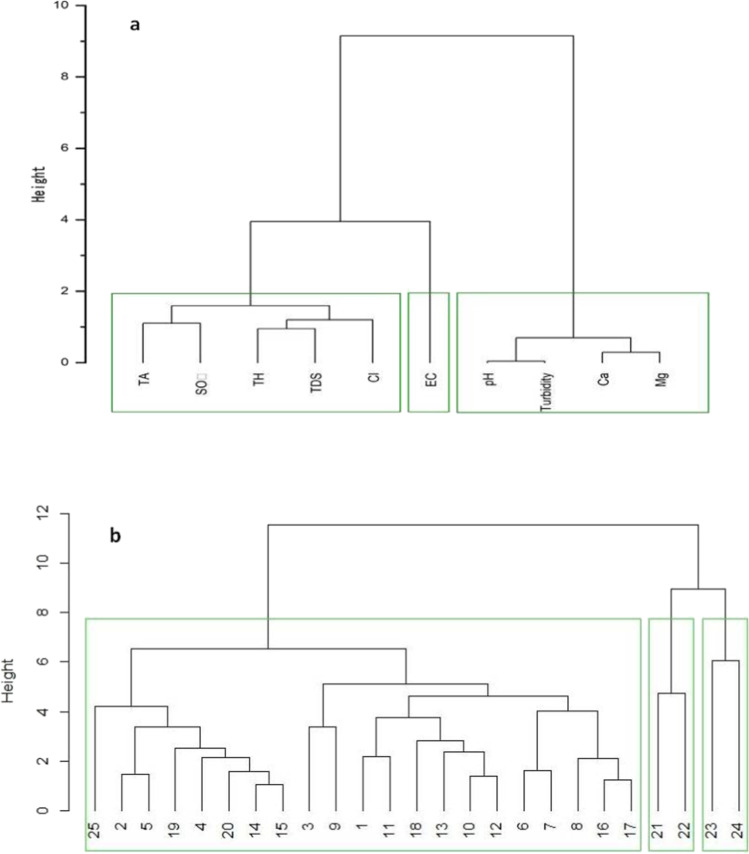
**a**  Dendrogram showing clustering of hydrochemical parameters. **b** Dendrogram showing clustering of sampling sites

For performing HCA, both R-mode and Q-mode HCA have been used to produce a hierarchy of clusters. These techniques have been applied to develop and merge homogeneous group of water samples into significant clusters and also to ascertain spatial similarity and location clustering within the sampling stations. Moreover, the clustering has been accomplished using Ward’s linkage criterion, and the results are illustrated in the form of a 2-D plot called dendrogram. The best number of clusters for our dataset has been determined using the NbClust package for both R mode and Q mode clustering techniques. The R-mode cluster analysis executed on groundwater samples produces three clusters (Fig. 8a). Cluster 1 includes TH, TDS, TA, SO_4_, Cl^−^, and it reflected the effect of saltwater intrusion and lateral flow from adjacent aquifer. Cl^−^ present in this cluster indicates surface contaminants, agricultural activities like fertilizer use and flushing of evaporated minerals from sedimentary rocks (Jiang et al. 2009). Cluster 2 contains EC, elucidated by salinity factor due to mineral dissolution. Cluster 3 consists of pH, turbidity, Ca and Mg, which might be defined by natural processes like severe evaporation, weathering of rich minerals and anthropogenic activities like agronomic practices, sewage activities and waste water discharge from industries. Q-mode cluster analysis performed on 25 sampling locations retains three clusters (Fig. 8b). Cluster 1 comprises 21 sampling sites which are S1–S20 and S25. Clusters 2 and 3 both contain two sampling sites which are S21–S22 and S23–24. The small Euclidean distance between Clusters 2 and 3 indicates that the water quality features of the sampling stations in these clusters are almost identical. The Euclidean distance of Cluster 2 and 3 is more than Cluster 1 which signifies high variability of water quality within these clusters. Cluster 3 sites are characterized by saltwater intrusion due to over exploitation and further highlight the influence of solubilization in the aquifer. The Cluster 2 sites are influenced by domestic and industrial effluents, whereas the sites at Cluster 1 indicate the influence of groundwater contamination via fertilizer leaching and runoff.

The discriminant analysis has been executed using the water quality parameters as predictors of membership in groundwater quality groups produced by HCA. A total of three discriminant functions were created from DA, by adopting stepwise DA for three significant variables (pH, EC and Cl) as shown in Table 7.

**Table 7:** Stepwise discriminant function coefficients

Parameters	DF_1_	DF_2_
EC	0.004	−0.002
pH	0.772	1.683
Cl	0.001	0.002
Constant	−12.117	−10.501

The value of DF coefficients highlights the importance of these variables. The higher the coefficient value of a DF shows, the higher the position of the variable in DA. The DF generated is given in the following equations:

12 $${\mathrm{DF}}_1=0.004\ \left[\mathrm{EC}\right]+0.772\left[\mathrm{pH}\right]+0.001\left[\mathrm{Cl}\right]-12.117$$

13 $${\mathrm{DF}}_2=-0.002\left[\mathrm{EC}\right]+1.683\left[\mathrm{pH}\right]+0.002\left[\mathrm{Cl}\right]-10.501$$

where DF_1_ and DF_2_ are the discriminant scores and EC, pH and Cl are the independent variables.

This shows that the primary contributing variables associated with the equation are EC, pH and Cl, which highlighted that these variables hold importance in terms of maintaining group differences. The statistical description of DA is presented in Table 8.

**Table 8:** Summary description of discriminant functions

Discriminant function	Wilk’slambda	Chi-square	Eigen value	P-level(Sig.)	Canonical correlation
DF_1_	0.018	84.845	20.478	0.000	0.976
DF_2_	0.378	20.347	1.646	0.000	0.789

**Fig. 9:**
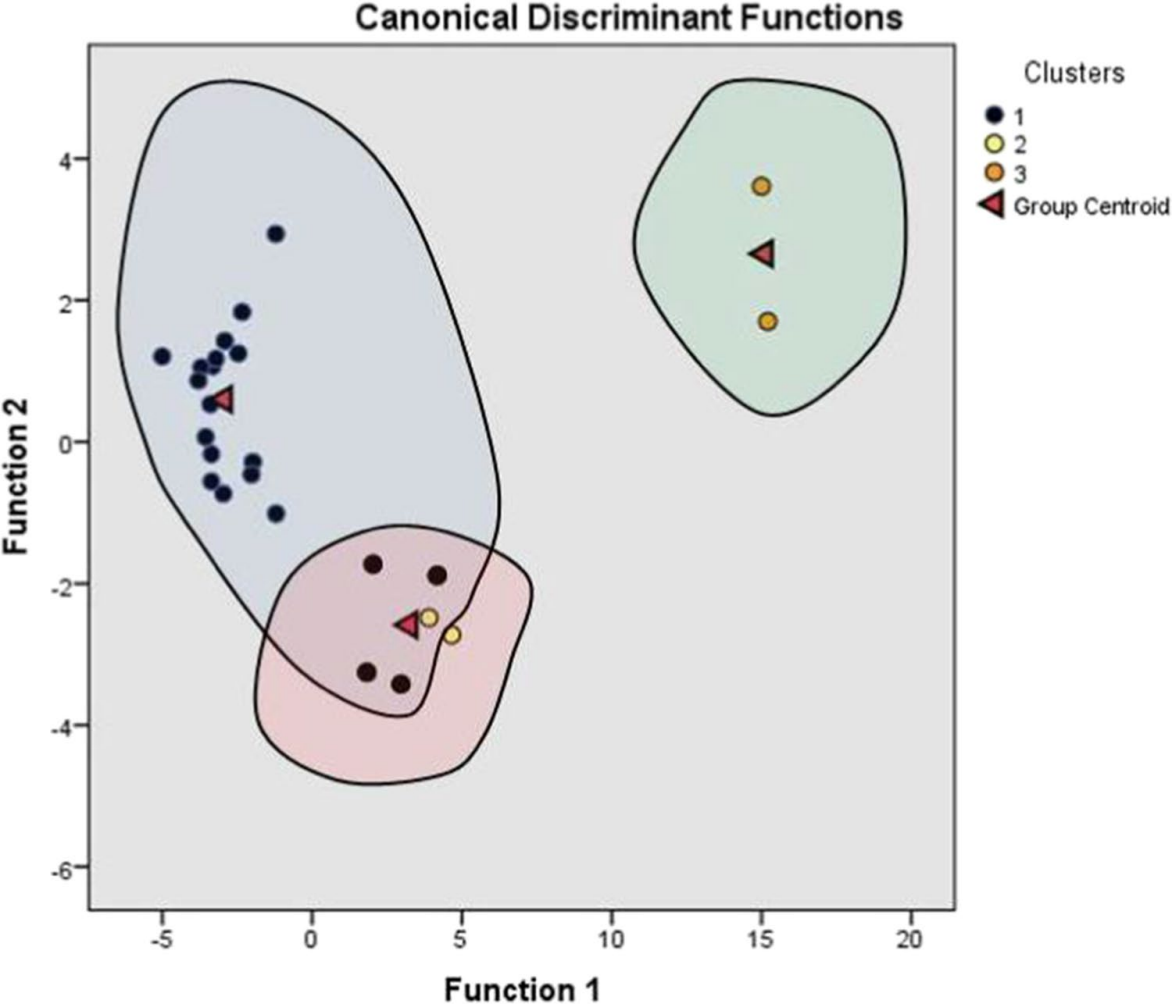
Scatterplot for the three water quality-based cluster groups in the space of two discriminant functions

In order to test the significance of discriminant function, Wilks’ lambda and chi-square distributions were adopted (Table 8). A small value of Wilks’ lambda and high chi-square value signify a greater discriminatory ability of the function, whereas high eigenvalues corresponding to high canonical correlation show usefulness of DF in differentiating between the cases. As shown in Table 8, the value of Wilk’s lambda and the chi-square for each DF varied from 0.018 to 0.378 and from 20.437 to 84.845, with a *p* value less than 0.01, suggesting that the spatial DA was reliable and effective. The scatterplot for all the observed values in the space of two DFs is shown in Fig. 9. The DA generates centroid for each cluster group. From Fig. 9, it is observed that the discrimination of groups and the distances between group centroids have been clearly represented.

## Conclusions

Groundwater salinization, aquifer depletion, intensive agricultural practices and associated fertilizer application are the major threats to groundwater sustainability in the Mewat region. This paper attempts to evaluate the potability of groundwater by the integrated use of WQIs, geostatistics and chemometric data analysis techniques. The study reveals that GWQI for the region varies from 21 to 278 with a mean value of 130, whereas the EWQI lies in the range of 104 to 276 with a mean value of 175. Based on the results of EWQI and GWQI analysis, it was observed that 72 and 64% of groundwater samples (*n* = 25) belong to the moderate (poor) to extremely poor quality domains respectively. The result for the semivariogram modeling shows that Gaussian model obtains the best fit for both EWQI and GWQI dataset. The chemometric study enabled us to elucidate the degrees and sources of groundwater contamination. HCA was helpful in classifying 25 sampling locations into three main clusters of similar groundwater characteristics. The dendrogram identified the governing factors of groundwater contamination and also depicted the worst affected regions where adaptive measures are needed to revive the groundwater quality. DA confirmed the clusters established by HCA and generated two DF that utilized three water quality parameters (EC, Cl^−^ and pH) to distinguish between these clusters. PCA was applied to discover the underlying factors and processes governing the groundwater chemistry. Three PCs were derived, which accounted for 69.81% of the total variance, respectively. Anthropogenic factors and geogenic processes (mineral dissolution, groundwater–rock interaction) were responsible for influencing the groundwater chemistry.

Although structured approaches based on aggregative WQI evaluation methods have been applied in the past, this study for the first time provides an intensive groundwater quality assessment of Mewat region by computing entropy weight coefficients for the water quality indicators. This numerical and graphical analysis of groundwater quality could function as a medium to apprise the bureaucrats and health and environmental activists about the water quality status of the region. This would further give impetus to the implementation of water protection techniques like community rainwater harvesting, direct surface and subsurface recharge that would help in restoring the regional groundwater resources.

## Nomenclature

*WQI*: Water quality index
*GWQI*: Ground water quality index
*EWQI*: Entropy water quality index
*GIS*: Geographic information system
*EC*: Electrical conductivity
*TDS*: Total dissolved solids
*pH*: Potential of hydrogen
*PCA*: Principal component analysis
*HCA*: Hierarchical cluster analysis
*DA*: Discriminant analysis
*PC*: Principal component
*TA*: Total alkalinity
*TH*: Total hardness
*CPCB*: Central pollution control board
*BIS*: Bureau of Indian Standards
*WHO*: World Health Organization
*NO*_*3*_^*−*^: Nitrate
*Cl*^*−*^: Chloride
*SO*_*4*_^*2−*^: Sulfate

## References

[CR1] Adak MD, Purohit KM, Datta J (2001). Assessment of drinking water quality of river Brahmani. J Environ Pollut.

[CR2] Alam M, Rais S, Aslam M (2012). Hydrochemical investigation and quality assessment of ground water in rural areas of Delhi, India. Environ Earth Sci.

[CR3] Alfaifi HJ, Kahal AY, Abdelrahman K, Zaidi FK, Albassam A, Lashin A (2020). Assessment of groundwater quality in Southern Saudi Arabia: case study of Najran area. Arab J Geosci.

[CR4] Amiri V, Rezaei M, Sohrabi N (2014). Groundwater quality assessment using entropy weighted water quality index (EWQI) in Lenjanat, Iran. Environ Earth Sci.

[CR5] APHA (2005) Standard methods for the examination of water and wastewater, 21st edn. Total organic carbon (TOC): high temperature combustion method (5310 A and 5310 B)

[CR6] Batabyal AK, Chakraborty S (2015). Hydrogeochemistry and water quality index in the assessment of groundwater quality for drinking uses. Water Environ Res.

[CR7] Bhuiyan MAH, Bodrud-Doza M, Islam AT, Rakib MA, Rahman MS, Ramanathan AL (2016). Assessment of groundwater quality of Lakshimpur district of Bangladesh using water quality indices, geostatistical methods, and multivariate analysis. Environ Earth Sci.

[CR8] BIS (Bureau of Indian Standards) (2012). Specification for drinking water IS 10500: 2012.

[CR9] Bureau of Indian Standards (BIS) 10500 (2012). Specification for drinking water.

[CR10] CPCB (2008). Guidelines for water quality management.

[CR11] Dalin C, Wada Y, Kastner T, Puma MJ (2017). Groundwater depletion embedded in international food trade. Nature.

[CR12] Doley B, Sivasami KS (2003). Trends in the quality of groundwater in Mewat Region, Gurgaon District, Haryana. Indian J Pub Admin.

[CR13] Duraisamy S, Govindhaswamy V, Duraisamy K, Krishinaraj S, Balasubramanian A, Thirumalaisamy S (2019). Hydrogeochemical characterization and evaluation of groundwater quality in Kangayam taluk, Tirupur district, Tamil Nadu, India, using GIS techniques. Environ Geochem Health.

[CR14] Durvey VS, Sharma LL, Saini VP, Sharma BK (1991). Handbook on the methodology of water quality assessment. Rajasthan Agriculture University, India, 156

[CR15] Egbueri JC (2020). Heavy metals pollution source identification and probabilistic health risk assessment of shallow groundwater in Onitsha, Nigeria. Anal Lett.

[CR16] Egbueri, J. C. (2020b) Groundwater quality assessment using pollution index of groundwater (PIG), ecological risk index (ERI) and hierarchical cluster analysis (HCA): a case study. Groundwater for Sustainable Development, 10, 100292.

[CR17] Egbueri JC (2021) Prediction modeling of potentially toxic elements’ hydrogeopollution using an integrated Q-mode HCs and ANNs machine learning approach in SE Nigeria. Environ Sci Pollut Res, 1-19 10.1007/s11356-021-13678-z 33774793

[CR18] Egbueri JC, Ameh PD, Unigwe CO (2020). Integrating entropy-weighted water quality index and multiple pollution indices towards a better understanding of drinking water quality in Ojoto area, SE Nigeria. Sci Afr.

[CR19] Egbueri JC, Ezugwu CK, Unigwe CO, Onwuka OS, Onyemesili OC, Mgbenu CN (2021). Multidimensional analysis of the contamination status, corrosivity and hydrogeochemistry of groundwater from parts of the Anambra Basin, Nigeria. Anal Lett.

[CR20] El Baba M, Kayastha P, Huysmans M, De Smedt F (2020). Evaluation of the groundwater quality using the water quality index and geostatistical analysis in the Dier al-Balah Governorate, Gaza Strip, Palestine. Water.

[CR21] Fatoba JO, Sanuade OA, Hammed OS, Igboama WW (2017). The use of multivariate statistical analysis in the assessment of groundwater hydrochemistry in some parts of southwestern Nigeria. Arab J Geosci.

[CR22] Gupta AK, Gupta SK, Patil RS (2003). A comparison of water quality indices for coastal water. J Environ Sci Health A.

[CR23] Harun HH, Kasim MRM, Nurhidayu S, Ash’aari ZH, Kusin FM, Karim MKA (2021). Association of physicochemical characteristics, aggregate indices, major ions, and trace elements in developing groundwater quality index (GWQI) in agricultural area. Int J Environ Res Public Health.

[CR24] Hatvani IG, Kirschner AK, Farnleitner AH, Tanos P, Herzig A (2018). Hotspots and main drivers of fecal pollution in Neusiedler See, a large shallow lake in Central Europe. Environ Sci Pollut Res.

[CR25] Helena B, Pardo R, Vega M, Barrado E, Fernandez JM, Fernandez L (2000). Temporal evolution of groundwater composition in an alluvial aquifer (Pisuerga River, Spain) by principal component analysis. Water Res.

[CR26] Islam ARMT, Ahmed N, Bodrud-Doza M, Chu R (2017). Characterizing groundwater quality ranks for drinking purposes in Sylhet district, Bangladesh, using entropy method, spatial autocorrelation index, and geostatistics. Environ Sci Pollut Res.

[CR27] Islam ARMT, Kabir MM, Faruk S, Al Jahin J, Bodrud-Doza M, Didar-ul-Alam M et al (2021) Sustainable groundwater quality in southeast coastal Bangladesh: co-dispersions, sources, and probabilistic health risk assessment. Environ Dev Sustain 1–30

[CR28] Jiang Y, Wu Y, Groves C, Yuan D, Kambesis P (2009). Natural and anthropogenic factors affecting the groundwater quality in the Nandong karst underground river system in Yunan, China. J Contam Hydrol.

[CR29] Jianhua W, Peiyue L, Hui Q (2011). Groundwater quality in Jingyuan County, a semi-humid area in Northwest China. Electron J Chem.

[CR30] Joarder MAM, Raihan F, Alam JB, Hasanuzzaman S (2008) Regression analysis of ground water quality data of Sunamganj District, Bangladesh

[CR31] Judeh T, Bian H, Shahrour I (2021). GIS-based spatiotemporal mapping of groundwater potability and palatability indices in arid and semi-arid areas. Water.

[CR32] Kazi TG, Arain MB, Jamali MK, Jalbani N, Afridi HI, Sarfraz RA (2009). Assessment of water quality of polluted lake using multivariate statistical techniques: a case study. Ecotoxicol Environ Saf.

[CR33] Li PH, Lee T, Youn HY (2020) Dimensionality reduction with sparse locality for principal component analysis. Math Probl Eng 2020

[CR34] Liu D, Liu C, Fu Q, Li T, Imran KM, Cui S, Abrar FM (2017). ELM evaluation model of regional groundwater quality based on the crow search algorithm. Ecol Indic.

[CR35] Liu F, Zhao Z, Yang L, Ma Y, Li B, Gong L, Liu H (2020). Phreatic water quality assessment and associated hydrogeochemical processes in an irrigated region along the Upper Yellow River, Northwestern China. Water.

[CR36] Machiwal D, Jha MK (2015). Identifying sources of groundwater contamination in a hard-rock aquifer system using multivariate statistical analyses and GIS-based geostatistical modeling techniques. J Hydrol Reg Stud.

[CR37] Machiwal D, Cloutier V, Güler C, Kazakis N (2018). A review of GIS-integrated statistical techniques for groundwater quality evaluation and protection. Environ Earth Sci.

[CR38] Mehra M, Oinam B, Singh CK (2016). Integrated assessment of groundwater for agricultural use in Mewat district of Haryana, India using geographical information system (GIS). J Indian Soc Remote Sensing.

[CR39] Mgbenu CN, Egbueri JC (2019). The hydrogeochemical signatures, quality indices and health risk assessment of water resources in Umunya district, southeast Nigeria. Appl Water Sci.

[CR40] Mukate S, Wagh V, Panaskar D, Jacobs JA, Sawant A (2019). Development of new integrated water quality index (IWQI) model to evaluate the drinking suitability of water. Ecol Indic.

[CR41] Naz A, Mishra BK, Gupta SK (2016). Human health risk assessment of chromium in drinking water: a case study of Sukinda chromite mine, Odisha, India. Exposure and Health.

[CR42] Nazir HM, Hussain I, Zafar MI, Ali Z, AbdEl-Salam NM (2016). Classification of drinking water quality index and identification of significant factors. Water Resour Manag.

[CR43] Omonona OV, Onwuka OS, Okogbue CO (2014). Characterization of groundwater quality in three settlement areas of Enugu metropolis, southeastern Nigeria, using multivariate analysis. Environ Monit Assess.

[CR44] Pant BR (2011). Ground water quality in the Kathmandu valley of Nepal. Environ Monit Assess.

[CR45] Pius A, Jerome C, Sharma N (2012). Evaluation of groundwater quality in and around Peenya industrial area of Bangalore, South India using GIS techniques. Environ Monit Assess.

[CR46] R Development Core Team (2007) R: a language and environment for statistical computing. R Foundation for Statistical Computing, Vienna, Austria. ISBN 3-900051-07-0, http://www.R-project.org

[CR47] Ramakrishnaiah CR, Sadashivaiah C, Ranganna G (2009). Assessment of water quality index for the groundwater in Tumkur Taluk, Karnataka State, India. Electron J Chem.

[CR48] Sadat-Noori SM, Ebrahimi K, Liaghat AM (2014). Groundwater quality assessment using the Water Quality Index and GIS in Saveh-Nobaran aquifer, Iran. Environ Earth Sci.

[CR49] Saha S, Reza AS, Roy MK (2019). Hydrochemical evaluation of groundwater quality of the Tista floodplain, Rangpur, Bangladesh. Appl Water Sci.

[CR50] Sarma VJ, Swamy AN (1981). Groundwater quality in Visakhapatnam basin, India. Water Air Soil Pollut.

[CR51] Sawyer CN, McCarty PL (1978). Chemistry for environmental engineering.

[CR52] Şener Ş, Şener E, Davraz A (2017). Evaluation of water quality using water quality index (WQI) method and GIS in Aksu River (SW-Turkey). Sci Total Environ.

[CR53] Sengani F, Zvarivadza T (2018). Assessment of groundwater quality: case study of Tshivhasa, Limpopo Province, South Africa. Symposium on Environmental Issues and Waste Management in Energy and Mineral Production.

[CR54] Shannon CE (1948) A mathematical theory of communication: The Bell System Technical Journal BSTJAN 0005-8580, 27, 379–423. Crossref Web of Science

[CR55] Sharma LM (2014). Innovation for making potable water available in saline groundwater areas. J Water Resour Prot.

[CR56] Solangi GS, Siyal AA, Babar MM, Siyal P (2019) Groundwater quality evaluation using the water quality index (WQI), the synthetic pollution index (SPI), and geospatial tools: a case study of Sujawal district, Pakistan. Hum Ecol Risk Assess Int J

[CR57] Todd DK (1980) Groundwater hydrology (p. 535). New York: Jon Wiley & Sons Inc

[CR58] Ukah BU, Egbueri JC, Unigwe CO, Ubido OE (2019). Extent of heavy metals pollution and health risk assessment of groundwater in a densely populated industrial area, Lagos, Nigeria. Int J Energy Water Resourc.

[CR59] Ukah BU, Ameh PD, Egbueri JC, Unigwe CO, Ubido OE (2020) Impact of effluent-derived heavy metals on the groundwater quality in Ajao industrial area, Nigeria: an assessment using entropy water quality index (EWQI). Int J Energ Water Res 1–14

[CR60] Usman UN, Toriman ME, Juahir H, Abdullahi MG, Rabiu AA, Isiyaka H (2014). Assessment of groundwater quality using multivariate statistical techniques in Terengganu. Sci Technol.

[CR61] Wada Y, Van Beek LP, Van Kempen CM, Reckman JW, Vasak S, Bierkens MF (2010) Global depletion of groundwater resources. Geophys Res Lett 37(20)

[CR62] WHO, World Health Organization (2011). Guidelines for drinking water quality.

[CR63] Yadav KK, Gupta N, Kumar V, Choudhary P, Khan SA (2018). GIS-based evaluation of groundwater geochemistry and statistical determination of the fate of contaminants in shallow aquifers from different functional areas of Agra city, India: levels and spatial distributions. RSC Adv.

[CR64] Zhou Y, Wang Y, Li Y, Zwahlen F, Boillat J (2013). Hydrogeochemical characteristics of central Jianghan Plain, China. Environ Earth Sci.

